# Recapitulating Trafficking of Nucleosides Into the Active Site of Polymerases of RNA Viruses: The Challenge and the Prize

**DOI:** 10.3389/fmedt.2021.705875

**Published:** 2021-12-14

**Authors:** Yves Boulard, Stéphane Bressanelli

**Affiliations:** Université Paris-Saclay, CEA, CNRS, Institute for Integrative Biology of the Cell (I2BC), Gif-sur-Yvette, France

**Keywords:** RNA virus, polymerase, inhibitor, nucleoside analog drugs, hepatitis C virus (HCV), SARS-CoV-2, molecular dynamics simulations

## Abstract

Nucleoside analogs are very effective antiviral agents with currently over 25 compounds approved for the therapy of viral infections. Still, their successful use against RNA viruses is very recent, despite RNA viruses comprising some of the most damaging human pathogens (e.g., Coronaviruses, Influenza viruses, or *Flaviviridae* such as dengue, Zika and hepatitis C viruses). The breakthrough came in 2013–2014, when the nucleoside analog Sofosbuvir became one of the cornerstones of current curative treatments for hepatitis C virus (HCV). An analog designed on the same principles, Remdesivir, has been the first approved compound against SARS-CoV-2, the coronavirus that causes the current COVID-19 pandemic. Both of these nucleoside analogs target the RNA-dependent RNA polymerase (RdRp) (NS5B for HCV, nsp12 for SARS-CoV-2). RdRps of RNA viruses display a peculiar elaboration of the classical polymerase architecture that leads to their active site being caged. Thus, triphosphate nucleosides and their analogs must access this active site in several steps along a narrow and dynamic tunnel. This makes straightforward computational approaches such as docking unsuitable for getting atomic-level details of this process. Here we give an account of ribose-modified nucleoside analogs as inhibitors of viral RdRps and of why taking into account the dynamics of these polymerases is necessary to understand nucleotide selection by RdRps. As a case study we use a computational protocol we recently described to examine the approach of the NTP tunnel of HCV NS5B by cellular metabolites of Sofosbuvir. We find major differences with natural nucleotides even at this early stage of nucleotide entry.

## Introduction

Nucleoside analogs are very effective antiviral agents with currently over 25 compounds approved for the therapy of viral infections. Hallmarks of antiviral therapy thus include nucleoside analogs targeting the DNA polymerases of several viruses, prominently among them HIV and hepatitis B virus but also some herpesviruses. These drugs are very effective and have been prescribed with great success for decades (1). Somewhat surprisingly there was no approved nucleoside analog for therapy against RNA viruses until 2013. Yet RNA viruses comprise some of the most important human pathogens and (nearly) all encode a prime target for a nucleoside analog: The RNA-dependent RNA polymerase (RdRp) that synthesizes new viral RNA genomes in infected cells without going through a DNA intermediate. Accordingly, there has been extensive research into developing nucleoside analogs against major RNA viruses, for instance against flaviviruses such as dengue or Zika virus (2), against Ebola virus, or against threatening respiratory viruses such as Influenza viruses and Coronaviruses (3). The breakthrough for nucleoside analogs in RNA virus therapy came in 2013–2014, when Sofosbuvir became in a very short time one of the cornerstones of the new treatments that have now proved to be a cure for hepatitis C virus (HCV) (4).

In this perspective, we will first recap how researchers were brought to a new concept of nucleoside analog inhibitor for viral RdRp. We will then examine viral RdRps taking the HCV RdRp as a model and describe the salient points in their atomic structures that make them a peculiar target. Finally, we will illustrate how molecular simulations can help understand currently mysterious modes of action of RdRp inhibitors developed on this new concept.

### 3'-Hydroxyl (ribo)Nucleotide Analogs as “Non-obligate” Chain Terminators of Viral RdRps

#### The Previous, and Misleading, Successes Against Viral DNA Polymerases: 3'-Deoxy Nucleoside Analogs

Effective nucleoside analogs targeting viral DNA polymerases (e.g., the HIV or HBV reverse transcriptases or the herpesvirus DNA polymerase) are chain terminators. They lack a normal 3'-hydroxyl ribose, e.g., Lamivudine (2',3'-dideoxy-3'-thiacytidine) that is used for the treatment of HIV-1, HIV-2, and HBV as it is effective against all three reverse transcriptases of these viruses (1). Hence after incorportation into the nascent viral DNA strand these 3'-deoxy analogs cannot attack the 5'-triphosphate of the next nucleotide, thus terminating DNA synthesis. Thus, an adaptation of the same strategy to RNA viruses seemed promising, i.e., 2'-hydroxy,3'-deoxy analogs (5).

#### The Shift in Perspective for Viral RdRps Exemplified by Sofosbuvir, an Effective Anti-HCV Drug

**Figure 1:**
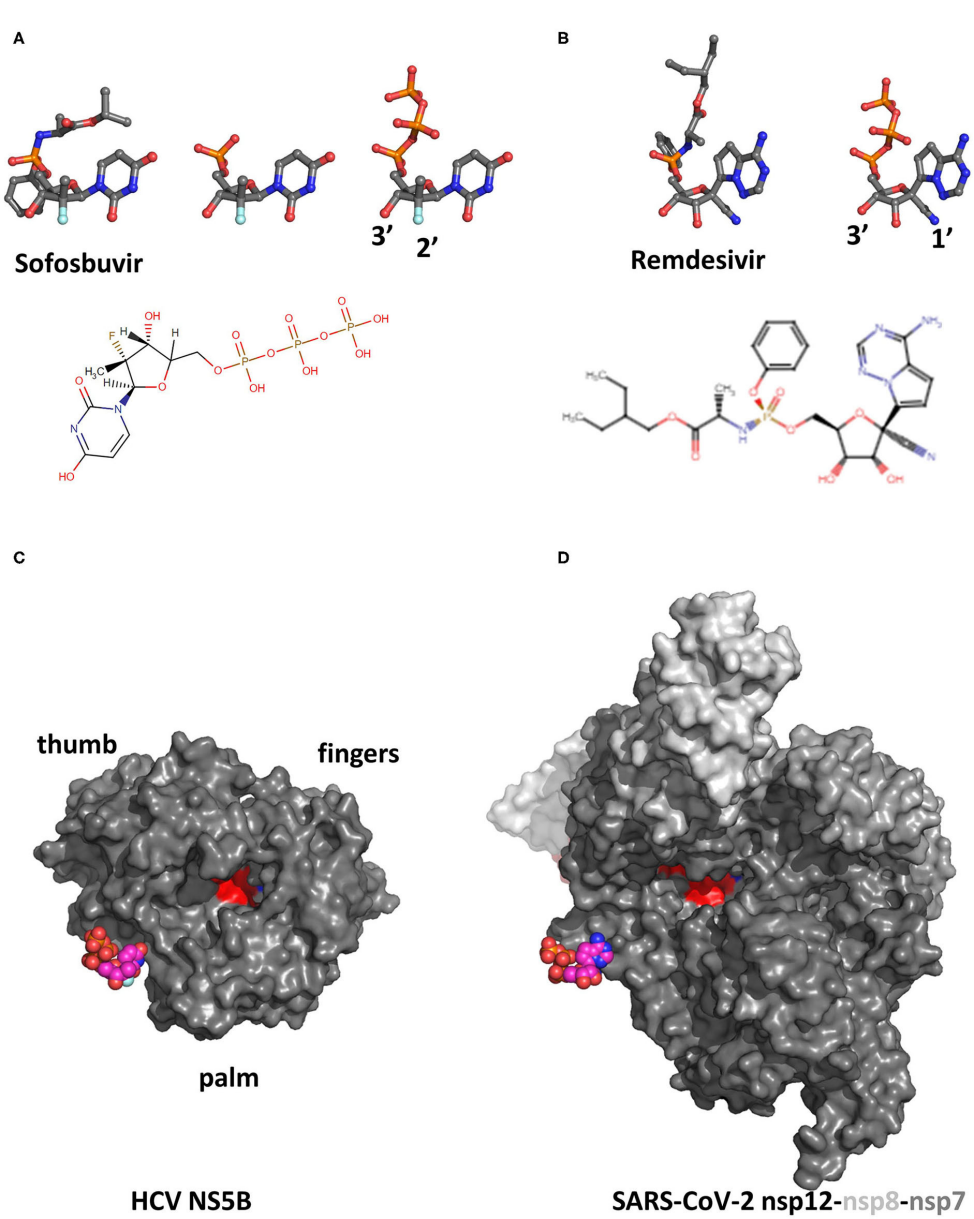
**(A)** Structure of Sofosbuvir (left) and of its successive monophosphate (middle, GS-606965) and triphosphate (right, GS-461203) metabolites. The chemical structure of GS-461203, the active form of Sofosbuvir, is also shown (downloaded from https://go.drugbank.com/metabolites/DBMET01383). Note the 2'-deoxy, 2'-fluoro, 2'-C-methyl substitution compared to uridine. **(C)** Structure of Remdesivir (left) and of its active triphosphate metabolite (right). The chemical structure of Remdesivir is also shown (downloaded from https://go.drugbank.com/drugs/DB14761). Note the 1'-cyano substitution compared to adenosine. Note also that both analogs harbor a normal 3'-hydroxyl **(A,B)**. For clarity, hydrogens are not shown in stick models. **(C,D)** Views of the triphosphate metabolites of Sofosbuvir outside the NTP tunnel of the HCV NS5B RdRp **(C)** and of Remdesivir outside the NTP tunnel of the SARS-CoV-2 nsp12-8-7 RdRp complex **(D)**. The proteins are shown in surface representation with active sites colored red.

Surprisingly, in screens of nucleoside analogs against intracellular replication of HCV that became available with the HCV replicon system (6), what emerged was actually 2'-modified, 3'-hydroxyl analogs. These were then shown genetically and biochemically to indeed inhibit the HCV RdRp, NS5B (7). 3'-hydroxyl analogs were thus termed “non-obligate” chain terminators for HCV NS5B and by extension for viral RdRps. Thorough biochemical characterization of their mode of action showed that despite their intact 3'-hydroxyl, 2'-modified nucleosides could cause impairment of elongation of the nascent RNA strand by NS5B (8). One of them is Sofosbuvir (Figure 1A, left), whose prosthetic groups are hydrolyzed *in vivo* to yield a UMP analog GS-606965 (Figure 1A, middle) that is converted by cellular kinases to the triphosphate metabolite of Sofosbuvir GS-461203 (Figure 1A, right and bottom). GS-461203 is thus a UTP analog and it is efficiently incorporated into nascent RNA by NS5B. Incorporation efficiency is about 1/45th that of UTP, which is very good for a nucleotide analog. The 2'F-2'C-methyl modification abolishes incorporation of the next nucleotide (nucleotide i+1), by an as yet unknown mechanism, i.e., GS-461203 is a *bona fide* chain terminator for NS5B (8). Sofosbuvir was the first of the all-oral, pan-genotypic, direct-acting antivirals that now constitute an effective cure for HCV infection (9).

#### Another Prominent Example: Remdesivir as a Tentative Drug Against Several RNA Viruses, Including Ebola Virus and SARS-CoV-2

Capitalizing on the grounbreaking success of Sofosbuvir, researchers, particularly in pharmaceutical companies, sought to develop nucleoside analogs as RNA virus antivirals designed on the same principles: Modified ribose or base, but intact 3'-hydroxyl. Remdesivir, a 1'-modified adenosine analog (Figure 1B), was thus developed first as a propective anti-HCV candidate. Then there were reports that although not useful against HCV, it was a broad-spectrum antiviral with activity in cell culture and small animal models against other single-stranded RNA viruses, both negative-sense (Ebola and Marburg viruses) (10) and positive-sense (Coronaviruses) (11). Indeed, Remdesivir was particularly scrutinized as a possible repurposed drug against SARS-CoV-2, the Coronavirus causing the current COVID-19 pandemic, and given emergency approval by the FDA and EMA. Subsequent clinical trials show little if any benefit in patients treated with Remdesivir compared to control groups (12). This is likely due to the fact that by the time Remdesivir is administered (i.e., by intravenous injection in hospital settings on the most serious COVID-19 cases), viral replication is already plummeting anyway (13).

At any rate, the mode of action of Remdesivir shed further light on the activity of non-obligate chain terminators of viral RdRps. The active (triphosphate) form (Figure 1B, right) is very efficiently incorporated into nascent RNA by the coronavirus RdRp. Indeed, it is one of the few nucleotide analogs that is actually more efficiently incorporated than the natural nucleotide it mimics, with an incorporation efficiency of about 3 times that of ATP for the SARS-CoV-2 nsp12-8 complex (14). Two very interesting observations are: (i) The analog's incorporation at position i causes a delayed chain termination, i.e., not at the next nucleotide incorporation (i+1) but at the third next (nucleotide i+3); (ii) Chain termination can be overcome by higher concentrations of incoming nucleotide i+3 (14). Hence the triphosphate metabolite of Remdesivir is a non-obligate chain terminator in these two senses for SARS-CoV-2 nsp12-8.

### Nucleotide Selection: The Peculiar “Fingertips” and Consequent “NTP Tunnel” of RdRps

These observations on Sofosbuvir and Remdesivir, and more generally on non-obligate chain terminators of viral RdRps, beg the question of the mechanisms by which these enzymes select incoming nucleotides. To consider this, we must first highlight key features of viral RdRp structure.

#### Elaboration on the “Right Hand” Architecture of A-Family Polymerases

Viral RdRps are related to a family of polymerases that notably also includes the so-called bacterial “A-family” DNA and RNA polymerases (i.e., DNA-dependent polymerases) and the reverse transcriptases of retroviruses such as HIV-1 (i.e., DNA polymerases that can use both RNA and DNA as templates) (15). Their structures can be likened to a right hand with subdomains of “fingers” and “thumb” on either side of the “palm” subdomain that harbors the two divalent metal ions (usually magnesium) that actually catalyze the addition of the next nucleotide onto the 3' end of the nascent nucleic acid strand. A surprising finding of the crystal structure of HCV NS5B was that, in contrast to A-family polymerases and reverse transcriptases, the fingers and thumb are actually connected by an additional substructure emanating from the fingers, which we termed the “fingertips” (16). As a result, the catalytic site with its two magnesium ions is completely encircled (17), leaving only a narrow tunnel of access for incoming nucleotides (Figure 1C). This surprising feature has since been found in all viral RdRps (18). This has held true for RdRp encoded by RNA viruses whose genome is double-stranded (19) or single-stranded, and in the latter case if it is positive-sense (i.e., it mimics a messenger RNA), for instance for the Coronavirus RdRp (20) or negative-sense (it is the complementary strand to messenger RNAs), for instance the influenza virus RdRp (21).

#### The NTP Tunnel as a Magnesium-Dependent Nucleotide Triage Belt

The outer parts of the nucleotide (NTP) tunnel are quite variable in different RdRps (Figures 1C,D). Particularly the loops on either side of the entrance are very variable and can harbor insertions of considerable size (22) and/or bind other subunits of the RNA synthesis complex. The tunnel itself is both very narrow, barely fitting the triphosphate form of nucleotides (23), and dynamic, as seen for instance in different crystal structures of the same RdRp with or without substrates bound (24). Simulation approaches relying on e.g., MM/GBSA to estimate free energy of binding from complexes with nucleotides (25) fail to consider the contributions of intermediate states along the tunnel. This may be the reason why Pan et al. paradoxically found a lower free energy of binding to HCV NS5B for GS-461203 than for UTP. Pan et al. also sought to describe the unbinding of these substrates along the NS5B NTP tunnel using steered molecular dynamics simulations (SMD) (25). This approach has however major disadvantages in the RdRp's case. Particularly the exit route may not be the same as the entry route, and the dynamic loops surrounding and extending into the NTP tunnel rearrange as the substrates enter (24). To clarify the mechanisms of nucleotide selection by RdRps, we recently published a series of molecular dynamics simulation protocols aiming at describing in atomic detail ribonucleotide entry along the NTP tunnel of HCV NS5B (26). We concluded that (i) The relevant initial complex is without either catalytic ion bound, this being essential for the opening of the tunnel entry. (ii) A nucleoside triphosphate site resides at the tunnel entrance, allowing the incoming nucleotide together with a first magnesium ion to first bind by its triphosphate moiety, then flip to engage its base into the tunnel (iii) The second magnesium ion is then a critical factor in modulating the local structure and dynamics in the tunnel, allowing the base of the incoming nucleotide to pass a gatekeeper residue and interrogate the template RNA strand. Notably, interactions of the nucleotide along the tunnel involve mostly the triphosphate and base, with interactions to the ribose depending on the incoming nucleotide.

#### Aspartate Stories: The Open Question of Ribose Recognition and Discrimination

In the case of the pyrimidine UTP, we noted that the ribose 3'-OH made an interaction with aspartate 220 of HCV NS5B (26). This residue is the first aspartate in the first motif ('motif A') of A-family-like polymerases (15) and in RdRp it has been shown to be critical for active site closure around the two magnesium ions and coordination of these ions for catalysis (24). In RdRp motif A is invariably DXXXXD, i.e., the fifth residue after the counterpart of HCV NS5B D220 is always an aspartate, whereas in reverse transcriptases, that use deoxyribonucleotides, it is DXXXXF/Y. It was thus assumed, not only that D225 was involved in ribose recognition, but that it served to discriminate between 2'-hydroxyl and 2'-deoxy nucleotides by direct interaction to the 2'-OH in the ribose (16). However, crystal structures of RdRp with incoming nucleotides have shown that D225's involvement is indirect: It serves instead in positioning a conserved serine that makes the actual hydrogen bond to ribonucleotides upon active site closure (24). Particularly crystal structures of HCV NS5B with template/primer RNA and incoming nucleotides, including Sofosbuvir, show that this hydrogen bonding network is labile and dispensable for active site closure (27). Our own work (26) shows that D225 is involved in reshaping the NTP tunnel in the early stages of nucleotide entry, leading to base engagement into the tunnel, before it flips back toward the active site. Thus, the whole of motif A has functions well-upstream of active site closure and catalysis in guiding nucleotide to the active site. How ribose is sensed remains however mysterious.

#### The Mitochondrial RNA Polymerase and the Crucial Issue of Non-toxicity

Several ribose-modified, particularly 2'-modified, analogs with excellent anti-HCV activity in cell culture were discontinued in phase I clinical trials due to severe toxicity issues (28). The major molecular determinant of this toxicity was pinpointed, not to nuclear RNA polymerisation, but to the mitochondrial RNA polymerase (POLRMT) (29). This A-family polymerase of bacterial origin was shown to be capable of incorporating the triphosphate metabolite of most of the nucleoside analogs, with subsequent chain termination. Strikingly, in 2'-methyl analogs, a single atom change, namely a 2'-fluor in place of the 2'-hydroxyl, could abrogate this off-target effect, as was achieved with Sofosbuvir (Figure 1A). There is thus a triple requirement for an effective drug against an RNA virus based on the concept of the non-obligate chain terminator: Efficient incorporation by the viral RdRp, effective chain termination, but also innocuity to POLRMT. Whether a particular ribose-modified non-obligate chain terminator can be a substrate of POLRMT is difficult to predict from the analog's structure alone (30). However, molecular dynamics simulations on phage T7 RNA polymerase, a close relative of POLRMT, have shed light at how the ribose may be recognized prior to nucleotide insertion into the catalytic site (31). The process would be dynamic and transient, which could explain the POLRMT sensitivity to some ribose-modified nucleotides, and it would involve structural elements that are not present in viral RdRp, which could explain how some others can still meet the three requirements of a non-obligate chain terminator.

### Case Study: Comparison of “Sofosbuvir triphosphate” (GS-461203) and “Sofosbuvir monophosphate” (GS-606965) With UTP Entering HCV NS5B

**Figure 2:**
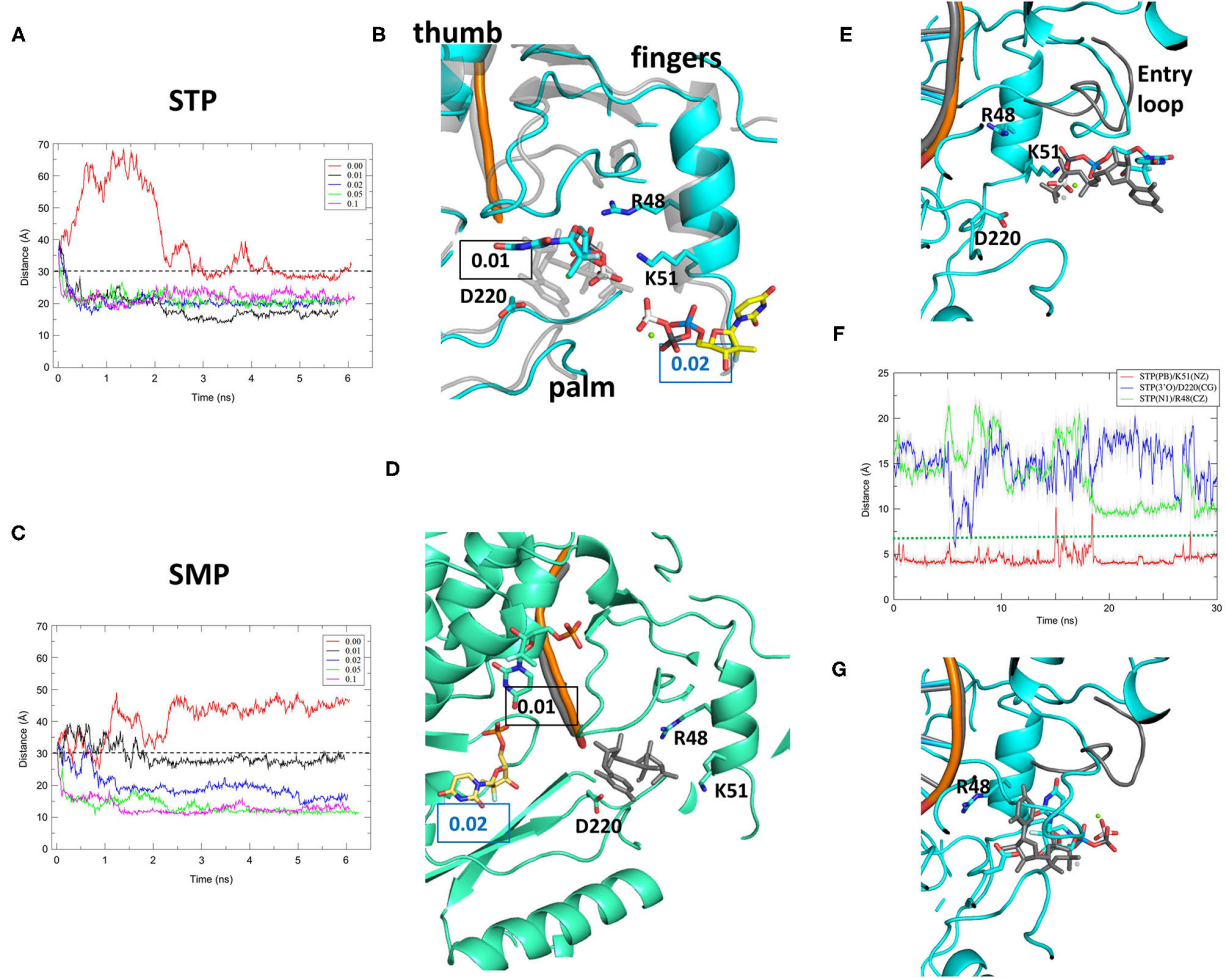
Simulations of the early steps of the entry of the triphosphate (STP) and monophosphate (SMP) metabolites of Sofosbuvir into the HCV NS5B NTP tunnel. **(A–D)** Molecular dynamics simulations with distance restraints on STP **(A,B)** and SMP **(C,D)**. **(A,C)** Evolution of the distances between centers of masses of the nucleotide and the NS5B active site for harmonic restraints of 0 (no restraint), 0.01, 0.02, 0.05, and 0.1 kcal/mol/Å^2^ (red, back, blue, green and pink curves, respectively). The dotted line indicates the distance of 30 Å at which the restraint drops to 0. **(B,D)** Final stable positions of STP and SMP for restraints of 0.01 (cyan for STP, greencyan for SMP) and 0.02 (yellow for STP, yelloworange for SMP; For clarity, only STP or SMP is shown) superimposed on a typical final position in our former work with UTP (transparent gray). The RNA backbone is colored orange. **(E–G)** Accelerated molecular dynamics simulations show spontaneous STP orientation at, but not engagement into, the NTP tunnel entry. **(E)** Initial snapshot of the simulations for STP (cyan) and UTP (dark gray; For clarity, only the RNA, entry loop and UTP with magnesium are displayed). View rotated by 90° from **(B)**. **(F)** Evolution of distances between the triphosphate, ribose and base of STP and residues K51, D220, and R48. Displayed are distances between STP Pβ and K51 Nζ (red curve), STP 3'O and D220 Cγ (blue curve), and STP N1 and R48 Cζ (green curve). The dotted green line indicates the smallest distance between UTP N1 and R48 Cζ in the accelerated simulation of our former work. **(G)** Snapshot at the end of the accelerated simulation (STP) or at the point nearest to R48 (UTP).

In order to compare entry of a successful 2'-modified, 3'-hydroxyl chain terminator to the natural nucleotide it mimics, we produced simulations of the early steps of entry into HCV NS5B of the triphosphate metabolite of Sofosbuvir GS-461203 (hereafter “STP”) with the exact same protocols we used for UTP and GTP (26). The first step is placing STP bound to a magnesium divalent cation well-outside the NTP tunnel, 40 Å away from the active site, and running a short simulation while harmonically restraining the distance between STP and the active site. There is thus a penalty for staying more than 30 Å away from the active site, but none below that limit. The STP simulations are remarkably like our formerly published UTP and GTP simulations: With a non-zero restraint STP together with its Mg^2+^ approaches NS5B and stays well-below 30 Å, finally settling near the entry of the NTP tunnel in a few nanoseconds (Figure 2A). The favored binding region involves NS5B K51 interacting with the STP triphosphate moiety, and the STP final positions and NS5B local conformations are very similar to those we found in the former UTP simulations (Figure 2B). We next ran the same protocol on a negative control, namely the monophosphate metabolite of Sofosbuvir GS-606965 (hereafter “SMP”). We chose SMP as it is an intracellular metabolite of Sofosbuvir with no activity against NS5B and the same net charge as STP-Mg^2+^. The results are very different from those obtained with either UTP-Mg^2+^, GTP-Mg^2+^ (26) or STP-Mg^2+^ (Figures 2A,B). With SMP, the molecule may end as close as or even closer to the active site than the triphosphate metabolites, although it needs higher restraints with SMP (Figure 2C). However, examination of the trajectories shows that SMP binds at different basic patches on NS5B's surface or (with higher restraints) in pockets of the NTP tunnel far from NS5B K51 (Figure 2D). This result confirms that our distance-restrained MD protocol introduces little bias in the end result. The interaction with K51 we consistently find with three different NTP-Mg^2+^ is not found here with SMP, indicating that monophosphate nucleosides may be discriminated against at the earliest step of nucleotide entry.

To further explore at which point STP entry may start displaying differences with UTP and GTP entry, we proceeded to the next step in our nucleotide entry protocol, an accelerated MD simulation (see the “Methods” section). This method is used to achieve a conformational change in a reasonable simulation time, something that proved necessary for UTP in our former work. As a starting point we chose the final snapshot of an STP simulation that is almost superimposable with a former UTP simulation (Figures 2B,E). As for UTP, the STP triphosphate moiety remains bound to the basic patch centering on K51 (Figure 2F, red curve), while the ribose (Figure 2F, blue curve) and base (Figure 2F, green curve) explore the tunnel entry, finally snapping into a conformation where the base points into the tunnel and toward the active site (Figure 2G, compare to Figure 2E). There is a major difference with the former UTP simulations however: Here STP does not engage into the tunnel, as seen in the larger distance that remains between the STP base and NS5B R48, that lines the tunnel (Figure 2F, green curve). The difference with UTP is linked to a different behavior of the “entry loop” of the fingertips. This loop remained retracted in the UTP simulations, keeping the NTP tunnel accessible (26), but with STP it closes down upon the tunnel (Figure 2G). This difference is all the more remarkable that it was not observed with GTP in our former work. It suggests that the STP ribose modifications may be sensed as early as at the stage of engagement into the NTP tunnel, possibly contributing to its lower incorporation efficiency by NS5B (8).

## Concluding Remarks

RdRp of RNA viruses have a highly labile nucleotide selection system, depending entirely on a series of concerted local rearrangements, none of which involves the opening and closing of suddomains as in their relatives, the A-family polymerases. These local rearrangements occur right from the entry of the RdRp's unique NTP tunnel, where nucleosides triphosphate bind, then engage into the tunnel; They further regulate a preinsertion site that involves the specific motif F3 common to RdRp and reverse transcriptases, but absent from A-family polymerases; They finally extend down to the active site itself that closes in the transition from preinsertion to insertion. Each of these rearrangements is subtle and dynamic, but their combination allows the required selectivity for the cognate nucleotide to be added to the growing RNA strand. We illustrate here with STP and SMP entry that discrimination against modified NTP may start even at a very early step. Particularly ribose check seems to start very far from the active site, suggesting that in such a system ribose check is most likely a distributed series of events along the NTP tunnel rather than a single checkpoint at or near the active site, as in A-family polymerases. Advanced molecular modeling will continue to be necessary to have an accurate grasp of these complex and important phenomena.

## Methods

### Construction of the Molecular System

We used the same molecular system, based on PDB 4WTA, as the one used in our previous study with UTP and incoming Mg at position 3 (located at the entry of the tunnel and 40 Å from the active site) and without Mg at the catalytic site (26). We replaced the UTP by the triphosphate metabolite of Sofosbuvir (STP) or its monophosphate metabolite (SMP). Parameters for STP and SMP were calculated with RED server (https://academic.oup.com/nar/article/39/suppl_2/W511/2505837) located at the University of Picardie in France.

### Molecular Dynamics Simulations

All simulations were performed exactly as for UTP and GTP in our former work and are described in detail there (26). Briefly, the AMBER 14 suite of programs (32) was used throughout. The ff14SB force field was employed for the protein and the ff99bsc0_chi0L3 for the RNA. The starting structure was neutralized with Cl- anions and hydrated with TIP3P water molecules that extended 10 Å from any protein atom. The system was minimized using steepest descent algorithm and used to initiate molecular dynamics at 300K. The SHAKE algorithm was used to constrain the motion of hydrogen-containing bonds within a 2 fs time step to integrate the equations of motions. The cut-off distance for van der Waals interactions was set to 10 Å and the Ewald particle mesh was used for long-range electrostatic interactions. Production runs were performed on the equilibrated structure with the NPT ensemble using a 2 fs time step.

#### Distance Restrained MD

drMD simulations are conventional MD simulations with an added harmonic energy term if the distance between the STP or SMP center of mass and the active site is above a threshold distance that we fixed at 30 Å. For the location of the active site we used the center of mass of residues 220, 318, and 319. From STP/SMP starting position, we performed five simulations with force constants ranging from 0 to 0.1 kcal/mol/Å^2^.

#### Accelerated Molecular Dynamics Simulations

aMD (33) is a technique for exploring the conformational space of biomolecules that accelerates the state to state evolution of a system relative to normal molecular dynamics making therefore rare events accessible in reasonable simulation times. In aMD, a positive boosting potential, ΔV(r), is applied to the system if the potential energy drops below a certain energy threshold (E). We used the dual boosting potential of the AMBER implementation of aMD (iamd = 3). We estimated the Amber aMD input parameters for the boost to the total potential and the extra boost to the torsion potential (EthreshP, alphaP, EthreshD, and alphaD) by using the average energy values computed from a 4–12 ns unbiased simulation of our system and we used a boost factor of 0.2.

All simulations were analyzed and rendered using the AMBER (32) cpptraj module and PyMol (34).

## Data Availability Statement

The raw data supporting the conclusions of this article will be made available by the authors, without unduereservation.

## Author Contributions

YB produced and analyzed the MD simulations. SB analyzed the MD simulations and wrote the paper. All authors contributed to the article and approved the submitted version.

## Funding

This work was supported by Agence Nationale de la Recherche sur le Sida et les hépatites virales (France REcherche Nord & sud SIDA - hiv Hépatites: FRENSH). Funding for open access charge: Université Paris-Saclay, Programme de recherche exceptionnel COVID-19.

## Conflict of Interest

The authors declare that the research was conducted in the absence of any commercial or financial relationships that could be construed as a potential conflict of interest.

## Publisher's Note

All claims expressed in this article are solely those of the authors and do not necessarily represent those of their affiliated organizations, or those of the publisher, the editors and the reviewers. Any product that may be evaluated in this article, or claim that may be made by its manufacturer, is not guaranteed or endorsed by the publisher.
